# Supplementary material for: Proteomic Changes during the Dermal Toxicity Induced by Nemopilema nomurai Jellyfish Venom in HaCaT Human Keratinocyte
Source: Toxins (Basel). 2021 Apr 27;13(5):311. doi: 10.3390/toxins13050311 (PMC8146130; doi:10.3390/toxins13050311)
Supplement: Supplementary file 1 [file toxins-13-00311-s001.zip › toxins-1165337-supp final.pdf]

Supplementary Materials: Proteomic Changes during the Dermal Toxicity Induced by *Nemopilema nomurai* Jellyfish Venom in HaCaT Human Keratinocyte

Indu Choudhary, Duhyeon Hwang, Jinho Chae, Wonduk Yoon, Changkeun Kang and Euikyung Kim

Table S1. Proteins with significant fold changes identified from HaCaT cells treated with *N. nomurai* venom.

Spot No	Accession Number	Protein Name	*P* Value	Fold Change	Cellular Components	Molecular Function
**Proteins with Decreased Volume**						
2319	P18206	Vinculin	0.040	1.7	Plasma membrane, cytoskeleton	Actin binding, cadherin binding, structural molecule activity
1901	Q9Y6U3	Adseverin	0.017	1.8	Cytoskeleton, podosome	1-phosphatidylinositol binding, calcium ion binding, negative regulation of cell proliferation
1417	O95259	Potassium voltage-gated channel subfamily H member 1	0.016	1.9	Nucleus, endosome, plasma membrane	Voltage-gated potassium channel activity, ion channel binding
1989	Q9NQ38	Serine protease inhibitor Kazal-type 5	0.044	1.6	Extracellular region or secreted	Serine-type endopeptidase inhibitor activity
1956	P14210	Hepatocyte growth factor	0.026	3.9	Extracellular region, membrane	Growth factor activity, protein tyrosine kinase activity
1937	Q6ZMZ3	Nesprin-3	0.001	3.8	Endoplasmic reticulum, nucleus	Actin filament binding
1621	P00747	Plasminogen	0.039	4.3	Extracellular region	Apolipoprotein binding, chaperone binding
1649	P16234	Platelet-derived growth factor receptor alpha	0.041	3.0	Cytoplasm, nucleus, cytoskeleton	Chromatin binding, transcription coactivator activity, histone deacetylase binding
816	O43707	Alpha-actinin-4	0.035	2.9	Cytoplasm, cytoskeleton, nucleus	Actin binding, ion channel binding, transcription coactivator activity

1052	P54762	Ephrin type-B receptor 1	0.005	1.9	Plasma membrane, endosome	ATP binding, protein tyrosine kinase activity, transmembrane signaling receptor activity
1915	O00203	Ubiquitin carboxyl-terminal hydrolase 36	0.017	2.2	Nucleolus, cytoplasm	Cysteine-type endopeptidase activity, RNA binding
1793	Q9UBK2	Peroxisome proliferator-activated receptor gamma coactivator 1-alpha	0.045	1.6	Nucleus	Alpha-tubulin binding, RNA binding, transcription factor binding
2057	Q5T0W9	Protein FAM83B	0.008	1.7	Membrane, cytoplasm	Epidermal growth factor receptor binding, phosphatidylinositol 3-kinase regulatory subunit binding
1903	Q6ZN19	Zinc finger protein 841	0.006	2.0	Nucleus	DNA binding, metal ion binding
1420	Q6ZN30	Zinc finger protein basonuclin-2	0.020	2.6	Nucleus	DNA binding, DNA-binding transcription factor activity
1424	Q9Y6C2	EMILIN-1	0.004	2.7	Extracellular region	Extracellular matrix constituent conferring elasticity, integrin binding involved in cell-matrix adhesion
1792	Q9UMS6	Synaptopodin-2	0.047	2.5	Nucleus, cytoskeleton	Alpha-actinin binding, protein binding, bridging
1423	Q05397	Focal adhesion kinase 1	0.037	1.8	Cytoskeleton, plasma membrane	Actin binding, protein tyrosine kinase activity, protein tyrosine kinase activity
1266	Q12866	Tyrosine-protein kinase Mer	0.027	1.9	Membrane	ATP binding, transmembrane signaling receptor activity
1933	Q96NW4	Mismatch repair endonuclease PMS2	0.040	1.6	Nucleus	Activation of MAPK activity, epithelial to mesenchymal transition, mitotic cell cycle
2233	Q96M96	FYVE, RhoGEF and PH domain-containing protein 4	0.048	1.5	Cytoskeleton	Glucocorticoid receptor activity, DNA-binding transcription factor activity
1944	Q13342	Nuclear body protein SP140	0.023	2.5	Nucleus, cytoplasm	DNA binding, DNA-binding transcription factor activity,
1186	O14730	Serine/threonine-protein kinase RIO3	0.009	2.5	Cytoplasm	ATP binding, protein serine/threonine kinase activity, metal ion binding

1553	Q13563	Polycystin-2	0.027	2.0	Plasma membrane, endoplasmic reticulum	Alpha-tubulin binding, RNA binding, transcription factor binding
1421	O43182	Rho GTPase-activating protein 6	0.029	3.0	Nucleus	ATP binding, translation activator activity
**Proteins with Increased Volume**						
2250	O75330	Hyaluronan mediated motility receptor	0.013	8.2	Cell surface, cytoplasm	Hyaluronic acid binding
1562	P15144	Aminopeptidase N	0.003	4.4	Plasma membrane	Signaling receptor activity, metallopeptidase activity
1978	Q86VD1	MORC family CW-type zinc finger protein 1	0.007	4.3	Nucleus	Zinc ion binding
2020	P51784	Ubiquitin carboxyl-terminal hydrolase 11	0.029	1.7	Nucleus, cytoplasm	Thiol-dependent ubiquitin-specific protease activity, cysteine-type endopeptidase activity
1378	O00206	Toll-like receptor 4	0.021	1.6	Endosome, plasma membrane	Lipopolysaccharide receptor activity, signaling receptor binding
2080	Q8N392	Rho GTPase-activating protein 18	0.013	3.4	Cytosol, plasma membrane	GTPase activator activity
2514	Q86UV5	Ubiquitin carboxyl-terminal hydrolase 48	0.026	8.7	Nucleus, cytoskeleton	Cysteine-type endopeptidase activity, thiol-dependent ubiquitin-specific protease activity
1696	Q969V6	MKL/myocardin-like protein 1	0.005	2.8	Nucleus	Actin binding, transcription coactivator activity
1929	Q9Y4L1	Hypoxia up-regulated protein 1	5.34e-004	6.3	Endoplasmic reticulum	ATP binding, chaperone binding
1590	Q14587	Heat shock protein 105 kDa	0.044	6.0	Cytoplasm	Adenyl-nucleotide exchange factor activity, alpha-tubulin binding
1198	Q96FS4	Signal-induced proliferation-associated protein 1	0.021	3.6	Nucleus	GTPase activator activity, protein C-terminus binding
1555	Q5T7N2	LINE-1 type transposase domain-containing protein 1	0.018	1.6		Single-stranded RNA binding

1886	P15918	V(D)J recombination-activating protein 1	0.045	1.8	Nucleus	DNA binding, metal ion binding, ubiquitin protein ligase activity
710	A6NI28	Rho GTPase-activating protein 42	0.034	5.5		GTPase activator activity
2199	P21709	Ephrin type-A receptor 1	0.024	3.0	Plasma membrane	Transmembrane signaling receptor activity, protein kinase binding, ATP binding
1389	Q93033	Immunoglobulin superfamily member 2	0.017	3.3	Membrane	Hydrolase activity
2305	Q4V348	Zinc finger protein 658B	0.025	5.7	Endoplasmic reticulum, plasma membrane	Cholesterol binding, sterol transporter activity
1122	Q02156	Protein kinase C epsilon type	0.035	6.4	Plasma membrane, nucleus, cytoskeleton	Protein kinase C activity, calcium-independent protein kinase C activity
1491	P49736	DNA replication licensing factor MCM2	0.026	5.5	Nucleus	Enzyme binding, metal ion binding, helicase activity
1827	P35527	Keratin, type I cytoskeletal 9	0.001	3.8	Cytoskeleton, extracellular region, nucleus	Structural constituent of cytoskeleton
